# Simultaneous bilateral video-assisted thoracic surgery is safe and feasible for multiple primary lung cancers

**DOI:** 10.1186/s13019-024-02941-2

**Published:** 2024-07-12

**Authors:** Libing Yang, Chao Guo, Ye Zhang, Huizhen Jiang, Lian Ma, Hongsheng Liu, Shanqing Li

**Affiliations:** 1 Department of Thoracic Surgery, Peking Union Medical College Hospital, Beijing, China; 2 Department of Information Management, Peking Union Medical College Hospital, Beijing, China

**Keywords:** Multiple primary lung cancers, Simultaneous bilateral VATS

## Abstract

**Background:**

The treatment for bilateral synchronous multiple primary lung cancers (MPLC) remains challenging. Simultaneous bilateral video-assisted thoracic surgery (VATS) may be an optimal treatment with curative intent, but its safety and feasibility are controversial.

**Methods:**

One hundred and fifty-eight patients who underwent simultaneous bilateral VATS (simultaneous group) and 79 who underwent two-staged bilateral VATS (two-staged group) were included in this study. Their medical records were retrospectively reviewed and analyzed.

**Results:**

The majority of patients were female and non-smokers. The most common surgical plan was lobectomy and contralateral wedge resection in both groups. There was no significant difference in the postoperative complication rate between the simultaneous groups and two-staged group (13.3% vs. 11.4%, *p* = 0.73). Patients who underwent simultaneous bilateral resection had shorter hospital stays, shorter anesthesia time and less chest drainage compared with those who underwent two-staged resection. Advanced TNM stage, complicated surgical plan and aggressive lymph node resection were risk factors for postoperative complications in simultaneous bilateral VATS. Patients in two groups had similar overall survival and disease free survival (*p* = 0.2).

**Conclusions:**

Simultaneous bilateral VATS for bilateral lung nodule resection is as safe and feasible as two-staged bilateral VATS. Patients who underwent simultaneous bilateral resection had similar or even better outcomes compared to that of the two-staged group. Simultaneous bilateral VATS is potentially an optimal treatment option for patients with erarly cTNM stage and good physical condition.

**Supplementary Information:**

The online version contains supplementary material available at 10.1186/s13019-024-02941-2.

## Introduction

Lung cancer is the leading cause of morbidity and mortality worldwide [1, 2], and the incidence rate of synchronous bilateral lung nodules has increased steadily in recent years with improvements in diagnostic methods and surveillance mechanisms. A significant proportion of these cases progress to synchronous multiple primary lung cancers (MPLC) [3]. The mechanisms underlying MPLC pathogenesis are poorly understood, although aging population, environmental and lifestyle changes, and gene mutations have been implicated [3, 4].

Early diagnosis is crucial but it is challenging to distinguish benign and malignant nodules, as well as MPLC from intrapulmonary metastasis, based on radiological features and serum biomarkers [5]. Early surgical resection can be beneficial for patients with suspected MPLC compared to chemotherapy or radiotherapy [6–9]. However, the choice of surgical strategy and procedure remains controversial. Two-staged bilateral surgery was preferred in most medical centers on account of its safety and stability. In the last decades, continuous improvements in thoracic minimally invasive surgery have increased the frequency of simultaneous bilateral video-assisted thoracic surgery (VATS). Although simultaneous bilateral VATS is highly effective [10], it is considered invasive and riskier by many surgeons.

In this retrospective study, we evaluated the safety and feasibility of simultaneous bilateral VATS for the treatment of synchronous bilateral lung nodules.

## Methods

### Patients

Data of a consecutive series of patients who underwent bilateral lung resection for multiple lung nodules at the Department of Thoracic Surgery, Peking Union Medical College Hospital (PUMCH) from January 2017 to January 2023 was retrospectively reviewed. The study was approved by the Institutional Review Board of PUMCH. One hundred and fifty-eight patients underwent simultaneous bilateral VATS (simultaneous group) and 79 underwent planned two-staged VATS (two-staged group). The exclusion criteria were as follows: (1) younger than 18 years of age, (2) metastatic carcinoma, (3) mediastinal mass resection, esophageal repair and other thoracic operations conducted simultaneously, and (4) unplanned two-stage surgery.

All patients were evaluated preoperatively by systemic radiography, pulmonary function test, echocardiography, etc. PET-CT was performed on most patients before surgery – 121 patients from simultaneous group and 57 patients from two-staged group to assist clinical staging. 6 patients from simultaneous group and 2 patients from two-staged group received bronchoscopy and EBUS before surgery. Demographic and clinical variables were collected from the electronic medical records which was connected to our lung cancer database. The tumors were staged as per the revised TNM system (eighth edition) and the most advanced tumor was used for final cancer staging [11, 12]. The activity of daily living (ADL) scores were calculated for each patient by nurses at admission and discharge.

### Surgery

The selection of the surgical approach was made by a panel of principal surgeons who were not involved in this study. The choice of surgical procedure for each case was guided by a comprehensive assessment of tumor features, such as size, location, the proportion of ground-glass opacity, and the likelihood of malignancy, as well as patient factors including age, general health status, and pulmonary function. Patient preferences were also considered in the decision-making process. Lesions deemed not to be malignant were excluded from the surgical resection plan.

All patients received general anesthesia with double-lumen endotracheal tube intubation. Various minimally invasive surgical approaches were used, including uni-portal, bi-portal, and three-portal, mainly depending on the complexity of the surgery. VATS lobectomy, segmentectomy resection, wedge resection and lymph node dissection were conducted according to the NCCN guidelines. Sublobar resection, which included both wedge resection and segmentectomy, was chosen for nodules that were 2 cm or smaller and met at least one of the following conditions: adenocarcinoma in situ, ground-glass opacity (GGO) exceeding 50%, or a nodule doubling time of 400 days or more. In the case of sublobar resections, a stapling device was utilized to separate the intersegmental plane. Lobectomy generally involved systematic lymph node dissection, whereas sublobar resection often involved systematic lymph node sampling. For simultaneous bilateral surgery, the patients were turned to their opposite side and re-sterilized after the first-side operation was finished.

### Perioperative management

Prior to surgery, the patients were instructed to cease smoking and alcohol consumption, and given pulmonary function training. The patients could resume oral fluid intake six hours after surgery, followed by a normal diet. Early mobilization was encouraged. For postoperative pain management, we routinely prescribe nonsteroidal anti-inflammatory drugs (NSAIDs) as the first line of analgesia. We adopted a stepwise escalation as needed based on the patient’s reported pain levels. In cases where patients had special pain management requirements, the anesthesiologist would initiate patient-controlled analgesia (PCA) immediately postoperatively. The anesthesiologist would then make daily visits to the patient’s bedside to assess the patient’s condition and determine the optimal time to discontinue the PCA.

A bedside chest radiograph was performed on day 1 post-operation to evaluate lung retention and chest drainage status. The chest tube was removed when chest drainage was less than 200 ml/d and no obvious air leak or chylothorax was detected. Patients without obvious pneumothorax and pleural effusion were considered for discharge [13].

### Statistical analysis

The demographic and clinical variables of both groups were compared by Wilcoxon test, chi-square test or Fisher exact test as appropriate using R (Version 1.1.453). The choice of statistical tests was guided by the nature of the data and the hypotheses being tested. Continuous variables were compared using the Wilcoxon test due to their non-normal distribution, while categorical variables were analyzed using the Chi-square test or Fisher exact test, as appropriate.

Odds ratios were calculated using the epitools package in R to quantify the association between binary outcomes and exposures. To visually depict these odds ratios alongside their confidence intervals, we generated forest plots with Prism Version 9.2.0, offering a clear and transparent summary of our study’s results. The survival analysis was performed with R *survival* package. The proportional hazards assumption was verified using Schoenfeld Residuals method.

## Result

### Patients characteristics

A total of 237 patients were included: 158 underwent simultaneous bilateral VATS, while 79 underwent planned two-staged bilateral resection. The demographic and clinical characteristics of the two groups are summarized in Table 1. The median age of both groups was 58-years and most patients were female. Less than 20% of them were current smokers or had a history of cigarette smoking. There was no significant difference in the history of cardiovascular diseases or left ventricular ejection fraction (LVEF) between the two groups. The most common cardiovascular comorbidities were hypertension, coronary heart disease and atrial fibrillation. Ten patients (6.3%) in the simultaneous group had a history of respiratory diseases including chronic obstructive pulmonary disease (COPD), pulmonary tuberculosis and asthma, bronchiectasis. And 5 patients in the two-staged group had respiratory diseases. There was no significant difference of FEV1/FVC or FEV1 between two groups.

**Table 1:** Characteristics of patients

	Simultaneous group	Two-staged group	*p*-value
n	158	79	
Age, median [IQR], yr	58.0 [50.0, 64.0]	58.0 [50.5, 63.0]	0.50
Male, n (%)	39 (24.7)	21 (26.6)	0.874
Smoking history, n (%)			0.318
Current smoker	8 ( 5.1)	2 ( 2.5)	
Former smoker	12 ( 7.6)	10 (12.7)	
Nonsmoker	138 (87.3)	67 (84.8)	
Comorbidities, n (%)			
Cardiovascular	55 (34.8)	19 (24.1)	0.124
Respiratory	10 ( 6.3)	5 ( 6.3)	1.000
Other tumor	27 (17.1)	7 ( 8.9)	0.132
FEV1, median [IQR], L	2.6 [2.2, 3.8]	2.6 [2.2, 3.7]	0.95
Predicted FEV1/FVC, median [IQR]	77.9 [73.2, 81.3]	78.0 [73.6, 82.7]	0.78
LVEF, median [IQR], %	69.0 [66.2, 73.0]	68.0 [66.0, 71.0]	0.13

Data are presented as median (with interquartile ranges) for continuous variables and n (%) for categorical variables. *p* values were obtained from Wilcoxon test for continuous variables and Fisher test for categorical variables

### Surgery

The choice of surgery was made at the discretion of an experienced surgical team. In both groups, the majority of patients underwent lobectomy and contralateral wedge resection (L-W). The second most common surgery type was segmentectomy resection and contralateral wedged resection (S-W) in the simultaneous group, and bilateral wedged resection in the two-staged group. The most common minimally invasive surgical approach was uni-portal on one side and three-portal on the other, which corresponded to the most common surgical method of lobectomy or segmentectomy on one side and wedge resection on the other. There was no significant statistical difference in the minimally invasive surgical approach between the two groups. Among patients who underwent two-staged bilateral resection, the median interval between the first and the second surgery was 89 days. Patients in the simultaneous group experienced less anesthesia time compared to the two-staged group. The overall operative time, total blood loss, and the total number of lymph nodes resected were comparable between the two groups (Table 2).

**Table 2:** Comparisons of surgery procedure between the simultaneous group and the two-staged group

	Simultaneous group	Two-staged group	*p*-value
Surgery type, n (%)			**0.013**
L-L	7 ( 4.4)	8 (10.1)	
L-S	14 ( 8.9)	14 (17.7)	
L-W	65 (41.1)	33 (41.8)	
S-S	7 ( 4.4)	1 (1.3)	
S-W	35 (22.2)	6 (7.6)	
W-W	30 (19.0)	17 (21.5)	
VATS approach, n(%)			0.12
Bilateral uni-portal	25 (15.8)	15 (19.0)	
Bilateral bi-portal	7 (4.5)	3 (3.8)	
Bilateral three-portal	24 (15.2)	23 (29.1)	
Uni-portal + three-portal	95 (60.1)	34 (43.0)	
Uniportal + bi-portal	3 (1.9)	1 (1.3)	
Bi-portal + three-portal	4 (2.5)	3 (3.8)	
Total operative time, median [IQR], min	174.5 [144.0, 203.2]	181.5 [155.8, 203.5]	0.45
Total anesthesia time, median [IQR], min	235.0 [200.0, 273.0]	305.0 [252.0, 349.0]	**< 0.01**
Total blood loss, median [IQR], ml	100[50, 200]	110 [100, 150]	0.16
Total number of lymph node resected, median [IQR]	21.0 [11.0, 31.5]	22.0 [15.0, 28.2]	0.84

L-L: bilateral lobectomy; L-S: lobectomy and contralateral segmentectomy resection; L-W: lobectomy and contralateral wedge resection; S-S: bilateral segmentectomy resection; S-W: segmentectomy resection and contralateral wedge resection; W-W: bilateral wedge resection

Data are presented as median (with interquartile ranges) for continuous variables and n (%) for categorical variables. *p* values were obtained from Wilcoxon test for continuous variables and Fisher test for categorical variables

### Pathological characteristics

Among patients who underwent simultaneous resection, 77.8% had bilateral adenocarcinoma (Table 3). Other less common pathological type included squamous-cell carcinoma, adenosquamous carcinoma and carcinoid. The was no significant difference in the TNM staging of both groups. The majority of patients were classified as IA stage in both groups (84.1% vs. 92.4%). Eight patients in the simultaneous group were classified as stage III, while only 1 patient in the two-staged group was at this TNM stage. 32.3% of the patients in the simultaneous group and 58.2% of patients in the two-staged group had more than two lesions (Table 3).

**Table 3:** Comparisons of postoperative pathologic characteristics between the simultaneous group and the two-staged group

	Simultaneous group	Two-staged group	*p*-value
Pathology, n (%)			0.078
Bilateral adenocarcinoma / in situ	123 (77.8)	58 (73.4)	
adenocarcinoma / in situ + AAH	22 (13.9)	18 (22.8)	
adenocarcinoma carcinoma + squamous carcinoma	1 (0.6)	1 (1.3)	
carcinoid + AAH	2 (1.3)	0	
adenosquamous + AAH	3 (1.9)	0	
squamous carcinoma + AAH	2 (1.3)	0	
Bilateral AAH	5 (3.2)	2 (2.5)	
TNM stage, n (%)			0.611
Tis	8 (5.1)	2 (2.5)	0.572
IA	132 (84.1)	73 (92.4)	
IB	5 (3.2)	1 ( 1.3)	
IIA	2 (1.3)	1 ( 1.3)	
IIB	3 (1.9)	1 ( 1.3)	
IIIA	8 (5.1)	1 ( 1.3)	
Number of lesions, n (%)			< 0.001
2	107 (67.7)	33 (41.8)	
>2	51(32.3)	46 (58.2)	

Data are presented as n (%) for categorical variables. *p* values were obtained from Fisher test for categorical variables. AAH: Atypical adenomatous hyperplasia.

### Short-term outcomes

The rate of postoperative complications did not differ significantly between the two groups (*p* = 0.73). Twenty-one (13.3%) who underwent simultaneous bilateral resection had postoperative complications including air leak (11 cases), infection (3 cases), pneumothorax, atelectasis, pulmonary embolism, deep vein thrombosis of the lower extremity, cerebral infarction, acute gastric retention, re-operation because of massive bleeding. Eighteen patients (11.4%) in the staged group had postoperative complications which all happened after the first surgery. Complications included air leak (10 cases), atrial fibrillation (2 cases), chylothorax, localized subcutaneous hematoma, acute urinary retention, acute coronary syndrome, and infection. The simultaneous group had shorter hospital stays and less chest drainage than the two-staged group (Table 4). We also compared short-term outcomes between the simultaneous group and the two-staged group in patients who underwent anatomic lung resections (i.e. segmentectomy or lobectomy). Similar results were observed (Table S1).

**Table 4:** Comparisons of short-term outcomes between the simultaneous group and the two-staged group

	Simultaneous group	Two-staged group	*p*-value
Postoperative complication, n (%)	21 (13.3)	18 (11.4)	0.73
Total Hospital stays, median [IQR], d	6.0 [5.0, 8.0]	13.0 [11.0, 15.0]	**< 0.01**
Average postoperative hospital stays, median [IQR], d	4.0 [3.0, 5.0]	3.5 [3.0, 4.5]	**< 0.01**
Chest drainage left, median [IQR], ml	450.0 [202.0, 695.0]	450.0 [250.0, 762.5]	0.51
Chest drainage right, median [IQR], ml	350.0 [150.0, 700.0]	550.0 [365.0, 821.0]	**< 0.01**
Total chest drainage, median [IQR], ml	850.0 [565.0, 1305.0]	1020.0 [700.0, 1600.0]	**0.03**
Chest drainage duration left, median [IQR], d	3.0 [2.0, 3.0]	3.0 [2.0, 3.0]	0.42
Chest drainage duration right, median [IQR], d	2.0 [1.0, 3.0]	3.0 [2.0, 3.0]	0.22
ADL score at discharge, mean (SD)	75.8 (14.7)	77.5 (13.6)	0.53

Data are presented as median (with interquartile ranges) for continuous variables and n (%) for categorical variables. *p* values were obtained from Wilcoxon test for continuous variables, Fisher test for categorical variables, chi-square test for postoperative complications. Statistically significant *p* values (*p* < 0.05) are highlighted in bold

### Long-term outcomes

**Fig. 1:**
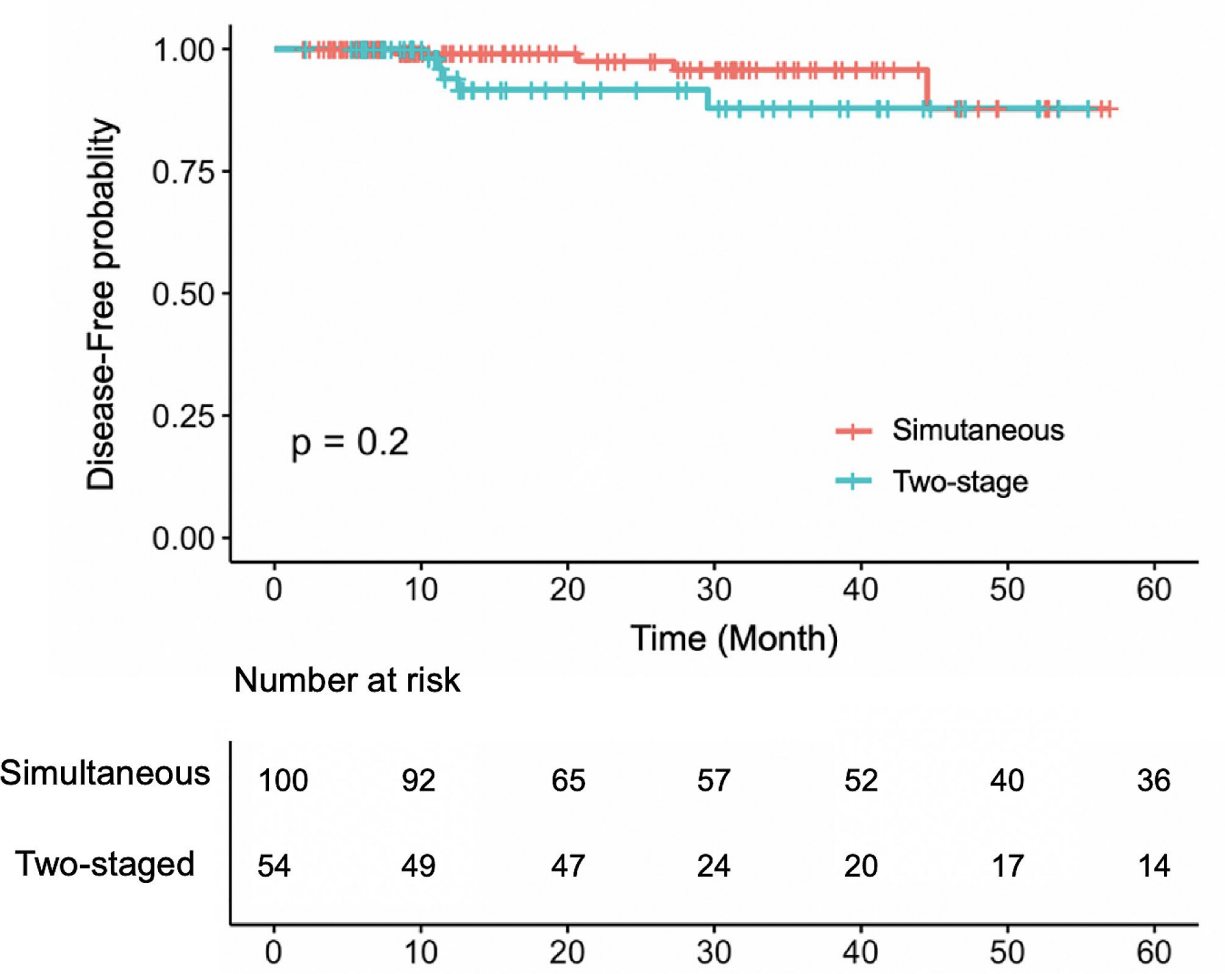
Kaplan-meier curve for survial. There was no significant difference of survial between patients (*p* = 0.2)

We used Cox-proportional hazards model to evaluate the association of two surgery strategies and survival rate. We did not observe significant difference between two groups (*p* = 0.2), but the simultaneous group appeared to have higher disease-free probability in the first 40 months (Fig. 1).

### Risk factors for postoperative complications

**Fig. 2:**
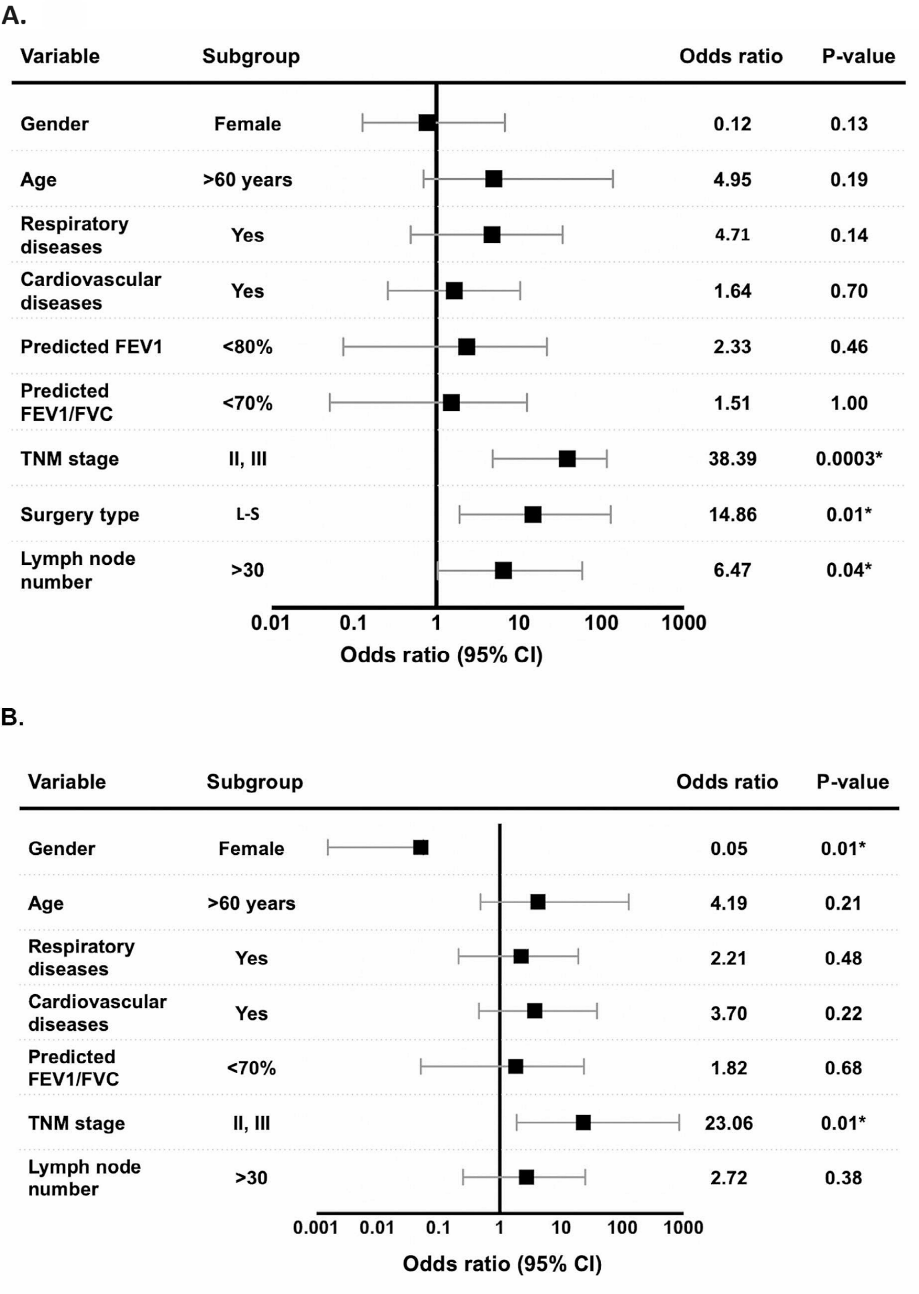
**A**) Risk factors for postoperative complications in the simultaneous group. TNM stage, surgery type and the number of lymph nodes removed are associated with postoperative complications. **B**) Risk factors for postoperative complications of simultaneous anatomic lung resections. Gender and TNM stage are associated with postoperative complications in patients who underwent simultaneous anatomic lung resections. *p < 0.05

TNM stage II and III, surgery type (L-S) and the number of resected lymph nodes (> 30) were significantly associated with postoperative complications following simultaneous bilateral VATS in all patients (Fig. 2A). We also focused on patients who underwent bilateral anatomic resections (i.e. segmentectomy or lobectomy), and found that male and advanced TMN stage would increase the risk of postoperative complications (Fig. 2B). Other factors such as age, co-morbidities, and pulmonary function did not significantly affect the risk of postoperative complications.

## Discussion

The landscape of lung cancer has evolved significantly over the past few decades. A particularly noteworthy trend is the increasing incidence of synchronous multiple primary lung cancers (MPLC), notably among women and individuals who have never smoked. The initial diagnostic criteria for MPLC were established by Martini and Melamed in 1975 [14]. These criteria were subsequently expanded upon by Antakli et al. [15] and Colice et al. in 1997 and 2003, respectively [16]. Subsequently, the American College of Chest Physicians (ACCP) issued guidelines in 2007 advocating for curative surgical resection in patients diagnosed with MPLC [17]. Since that time, no further guidelines or extensive studies concentrating on surgical strategies for MPLC have been published, only a few small-scale studies have focused on the treatment of MPLC [18].

Bilateral MPLC presents a more complex clinical challenge, requiring a comprehensive therapeutic strategy that delicately balances the extent of surgical resection to remove tumors while preserving lung function [19]. Additionally, physicians need to determine the optimal timing for surgery, taking into account the severity of the disease and the patient’s overall physical health, to ensure the best possible outcomes. Theoretically, simultaneous bilateral VATS is beneficial because it reduces the risk of cancer progression by reducing the duration of treatment. The second surgery of two-staged bilateral resection is usually performed several months after the first one (median 89 days in our study) to allow patients to recover. In the case of more invasive tumors, this interim period may lead to disease progression and even distant metastasis. Recent developments in minimally invasive surgery and enhanced recovery after surgery (ERAS) have made simultaneous bilateral VATS more feasible.

Thus, we aimed to explore the best surgical strategy for patients with bilateral MPLC. In this study, we retrospective compared the safety and feasibility of simultaneous bilateral VATS with that of planned two-staged bilateral VATS. As a real-world study, the surgical plans were made by a clinical team completely independent of this research. In theory, the lead surgeon took into account not only the patient’s tumor characteristics, but also other factors such as patients’ age, comorbidities, and lung function to minimize surgical risks. Fortunately, in our cohort, statistical analyses revealed that patients’ baseline characteristics including age, gender, cardiovascular comorbidities, respiratory system comorbidities, lung function, and heart function were well-matched across groups, with no significant differences. This largely eliminated the influence of selection bias on the study’s outcomes.

According to the randomized controlled study by the American Lung Cancer Research Group in 1995 [20], lobectomy is considered the standard surgical approach for the treatment of lung cancer. With the progression of minimally invasive surgical techniques and an increased understanding of early lung cancer, anatomic segmentectomy for the treatment of certain early-stage lung cancers has increasingly gained clinical acceptance. Researchers found that for patients with non-small cell lung cancer of less than 2 cm, the long-term survival with segmentectomy is not inferior to lobectomy, and compared to lobectomy, segmentectomy can better preserve lung function. Consequently, segmentectomy is gradually becoming the recommended approach for treating stage IA lung cancer [13, 21, 22]. In our cohort study, one-side lobectomy and contralateral wedge resection were the predominant surgical procedures in both the simultaneous and two-staged groups. For those undergoing simultaneous resection, segmentectomy on one side combined with contralateral wedge resection was the second most frequent approach. Additionally, in each group, there were over 20 patients undergone bilateral anatomical lung surgeries. Collectively, these findings indicate that the proportion of patients receiving at least one-side anatomical lung resection during simultaneous surgery is similar to that of the two-staged approach. We can conclude that the extent and complexity of surgical resection were not diminished by the simultaneous surgical plan. More importanly, the number of lymph nodes removed during simultaneous surgery was similar compared to that in two-staged surgery, indicating that the thoroughness of lymph node dissection was not influenced.

We also observed that patients could benefit from simultaneous surgical plan. Patients in the simultaneous group had shorter anesthesia time, potentially reducing the perioperative and anesthesia risks. Additionally, the simultaneous procedure helped to prevent readmission and shortened the overall hospital stay duration. These results consistent with the conclusions from previous researches [8, 10, 23–26].

Nevertheless, the safety and stability of simultaneous bilateral resection are still debated since it may cause greater surgical trauma and increase lung function loss. In our study, the rate of postoperative complications in the simultaneous group was 10.2% (6/59), which was similar to that observed in the two-staged group (8.5%, 5/58). There was one death in the simultaneous group due to massive blood loss during right segmentectomy resection after left lobectomy, and subsequent multiple organ failure in ICU. Postoperative pathological examination showed bilateral adenocarcinoma and the patient was staged as IIA. The postoperative complication rate in our study was lower compared to that found in other teams’ studies (14% ∼ 29%) [8, 10, 23–26]. In Dr. Yao’s research which included 29 patients who underwent simultaneous surgeries, the rate of postoperative complication was 21%, primarily air leaks and severe pneumonia [23]. In another study from Dr. Zhang’s team, postoperative complications was observed in 8 of 56 patients (14.2%) [24]. We also found that there was no significant difference in the average blood loss, chest drainage duration and ADL scale at discharge between the two groups. Interestingly, the volume of right-side chest drainage in the simultaneous group was significantly lower than that of the two-staged group. These results showed that the simultaneous bilateral VATS was as safe as two-staged bilateral VATS during in the perioperative period. Due to the significance of anatomical lung resection, we conducted a separate analysis of these patients and found that postoperative complications and short-term prognostic indicators were the same between the two groups. This confirms the safety of simultaneous surgery, even for complex bilateral procedures.

In order to select the optimal surgical procedure for each patient, it is essential to identify the risk factors for postoperative complications. A previous study on 41 patients reported that preoperative comorbidities, impaired pulmonary function and removal of more than 9 lung segments may lead to complications after simultaneous bilateral surgery [8]. A study from Dr. Liu’s team reported that bilateral lobectomy has higher postoperative complications compared to patients who underwent bilateral wedged resections [27]. Our study identified that TNM stage II and III, lobectomy and contralateral segmentectomy resection, and removal of > 30 lymph nodes were associated with a higher risk of postoperative complications in the simultaneous group. We did not observe similar associations in the two-staged group. In our study and others, more invasive and complicated surgery types were related to an increased risk of complications. And we further recommend that surgeons should be cautious in selecting simultaneous bilateral VATS for patients with larger lung nodules or possible lymph node metastasis detected during preoperative CT or PET/CT.

Our study also reported the results of long-term follow-up, a lightspot that many other studies did not include. We found that staged surgery or simultaneous surgery did not affect the long-term prognosis of patients, and even the survival rate of simultaneous patients showed a higher trend in the early period.

Our study has several limitations that ought to be considered. First, as a single center study, the findings cannot be extrapolated to the general population. Second, the retrospective design of our study may have led to selection bias. Third, although our cohort is representative of the largest data of simultaneous bilateral VATS nationwide, the sample size was still small. Fourth, although we have made efforts to follow up with patients for as long as possible, some patients still have a relatively short follow-up period. Further perspective studies with larger cohorts are needed to validate our findings. In addition to the classic bilateral intercostal approach, the subxiphoid single-port approach for simultaneous bilateral surgery is gradually being applied. Studies by Negi et al. [28]. and Wang et al. [29]. have respectively demonstrated the feasibility and advantages of subxiphoid single-port thoracoscopic simultaneous bilateral surgery, including the protection of intercostal nerves, reduction of postoperative pain, and acceleration of recovery. The subxiphoid single-port simultaneous bilateral surgery may represent a new direction for the future treatment of synchronous bilateral lung cancer and worth further research,

In conclusion, simultaneous bilateral VATS is as safe and feasible as two-staged bilateral VATS for bilateral lung nodules. Patients in the simultaneous group had similar or even better short-term outcomes compared to the two-staged group. Simultaneous bilateral VATS holds the potential as the optimal treatment for bilateral MPLC in patients with early TNM stage and better physical condition.

### Electronic supplementary material

Below is the link to the electronic supplementary material.

Supplementary Material 1

## Data Availability

No datasets were generated or analysed during the current study.

## References

[1] Barta JA, Powell CA, Wisnivesky JP. Global epidemiology of Lung Cancer. Ann Glob Health. 2019;85(1). 10.5334/aogh.2419 PMC6724220 30741509

[2] Bray F, Ferlay J, Soerjomataram I, Siegel RL, Torre LA, Jemal A (2018). Global cancer statistics 2018: GLOBOCAN estimates of incidence and mortality worldwide for 36 cancers in 185 countries. CA Cancer J Clin.

[3] Xue X, Liu Y, Pan L, Wang Y, Wang K, Zhang M (2013). Diagnosis of multiple primary lung cancer: a systematic review. J Int Med Res.

[4] Zheng R, Shen Q, Mardekian S, Solomides C, Wang ZX, Evans NR (2020). 3rd. Molecular profiling of key driver genes improves staging accuracy in multifocal non-small cell lung cancer. J Thorac Cardiovasc Surg.

[5] Zhao L, Liu C, Xie G, Wu F, Hu C (2020). Multiple primary lung cancers: a New Challenge in the era of Precision Medicine. Cancer Manag Res.

[6] Battafarano RJ, Meyers BF, Guthrie TJ, Cooper JD, Patterson GA (2002). Surgical resection of multifocal non-small cell lung cancer is associated with prolonged survival. Ann Thorac Surg.

[7] Peng Y, Ren W, Wang H, Li M, Feng Z, Peng Z (2017). Surgical treatment is an effective approach for patients with synchronous multiple primary lung cancers. J Cancer Res Ther.

[8] Zheng H, Peng Q, Xie D, Duan L, Zhao D, Jiang G (2021). Simultaneous bilateral thoracoscopic lobectomy for synchronous bilateral multiple primary lung cancer-single center experience. J Thorac Dis.

[9] Ishikawa Y, Nakayama H, Ito H, Yokose T, Tsuboi M, Nishii T (2014). Surgical treatment for synchronous primary lung adenocarcinomas. Ann Thorac Surg.

[10] Mun M, Kohno T (2007). Single-stage surgical treatment of synchronous bilateral multiple lung cancers. Ann Thorac Surg.

[11] Okada M, Tsubota N, Yoshimura M, Miyamoto Y (1998). Operative approach for multiple primary lung carcinomas. J Thorac Cardiovasc Surg.

[12] Goldstraw P, Chansky K, Crowley J, Rami-Porta R, Asamura H, Eberhardt WE (2016). The IASLC Lung Cancer Staging Project: proposals for revision of the TNM Stage groupings in the Forthcoming (Eighth) Edition of the TNM classification for Lung Cancer. J Thorac Oncol.

[13] Zhang J, Bai W, Guo C, Liu L, Wang G, Huang C (2020). Postoperative short-term outcomes between Sublobar Resection and Lobectomy in patients with Lung Adenocarcinoma. Cancer Manag Res.

[14] Martini N, Melamed MR (1975). Multiple primary lung cancers. J Thorac Cardiovasc Surg.

[15] Antakli T, Schaefer RF, Rutherford JE, Read RC (1995). Second primary lung cancer. Ann Thorac Surg.

[16] Colice GL, Rubins J, Unger M (2003). American College of Chest P. Follow-Up and surveillance of the lung cancer patient following curative-intent therapy. Chest.

[17] Shen KR, Meyers BF, Larner JM, Jones DR, American College of Chest P (2007). Special treatment issues in lung cancer: ACCP evidence-based clinical practice guidelines (2nd edition). Chest.

[18] Loukeri AA, Kampolis CF, Ntokou A, Tsoukalas G, Syrigos K (2015). Metachronous and synchronous primary lung cancers: diagnostic aspects, surgical treatment, and prognosis. Clin Lung Cancer.

[19] Jung EJ, Lee JH, Jeon K, Koh WJ, Suh GY, Chung MP (2011). Treatment outcomes for patients with synchronous multiple primary non-small cell lung cancer. Lung Cancer.

[20] Ginsberg RJ, Rubinstein LV (1995). Randomized trial of lobectomy versus limited resection for T1 N0 non-small cell lung cancer. Lung Cancer Study Group. Ann Thorac Surg.

[21] Altorki NK, Yip R, Hanaoka T, Bauer T, Aye R, Kohman L (2014). Sublobar resection is equivalent to lobectomy for clinical stage 1A lung cancer in solid nodules. J Thorac Cardiovasc Surg.

[22] Subramanian M, McMurry T, Meyers BF, Puri V, Kozower BD (2018). Long-term results for clinical Stage IA Lung Cancer: comparing Lobectomy and Sublobar Resection. Ann Thorac Surg.

[23] Yao F, Yang H, Zhao H (2016). Single-stage bilateral pulmonary resections by video-assisted thoracic surgery for multiple small nodules. J Thorac Dis.

[24] Zhang Y, Wang Y, Lv C, Shu X, Wang J, Yang Q (2018). Clinical analysis of 56 cases of simultaneous bilateral video-assisted thoracoscopic surgery for bilateral synchronous multiple primary lung adenocarcinoma. J Thorac Dis.

[25] Lin S, Yang C, Guo X, Xu Y, Wang L, Wang Z (2021). Simultaneous Uniportal video-assisted thoracic surgery of bilateral pulmonary nodules. J Cardiothorac Surg.

[26] Qu R, Hao Z, Zhang Y, Bie L, Fu X, Zhang N (2020). Single-center experience of simultaneous bilateral uni-portal video-assisted thoracoscopic surgery for multiple ground-glass opacities. J Cardiothorac Surg.

[27] Liu YW, Wu MH, Kao CN, Chiang HH, Lee JY, Li HP (2023). Lobectomy Versus Sublobar Resection in simultaneous bilateral thoracoscopic lung resection. World J Surg.

[28] Negi T, Suda T, Tochii S, Hoshikawa Y (2020). Subxiphoid uniportal bilateral lung wedge resection. Eur J Cardiothorac Surg.

[29] Wang J, Xu M, Zhang C, Wei D (2022). Clinical analysis of subxiphoid single-port thoracoscopic surgery for simultaneous bilateral lung lesion resection. BMC Surg.

