# Supplementary material for: Simultaneous bilateral video-assisted thoracic surgery is safe and feasible for multiple primary lung cancers
Source: J Cardiothorac Surg. 2024 Jul 12;19:436. doi: 10.1186/s13019-024-02941-2 (PMC11242011; doi:10.1186/s13019-024-02941-2)
Supplement: Supplementary file 1 — Supplementary Material 1 [file 13019_2024_2941_MOESM1_ESM.docx]

**Table S1. Comparisons of short-term outcomes between the simultaneous group and the two-staged group in patients who underwent bilateral anatomic lung resections.**

|  | **Simultaneous group** | **Two-staged group** |
| --- | --- | --- |
| Surgery type, n (%) | 28 | 23 |
| L-L | 7 ( 4.4) | 8 (10.1) |
| L-S | 14 ( 8.9) | 14 (17.7) |
| S-S | 7 ( 4.4) | 1 (1.3) |

|  | **Simultaneous group** | **Two-staged group** | **p-value** |
| --- | --- | --- | --- |
| Postoperative complication,  n (%) | 5 (17.9) | 5 (21.7) | 0.12 |
| Total Hospital stays,  median [IQR], d | 7.0 [5.0, 8.0] | 13.0 [11.0, 16.0] | **<0.01** |
| Average postoperative  hospital stays, median  [IQR], d | 4.0 [4.0, 6.0] | 3.5 [3.0, 5.0] | **0.05** |
| Chest drainage left,  median [IQR], ml | 600.0 [350.0, 805.0] | 460.0 [362.5, 710.0] | 0.53 |
| Chest drainage right,  median [IQR], ml | 450.0 [300.0, 700.0] | 530.0 [390.0, 800.0] | 0.29 |
| Total chest drainage,  median [IQR], ml | 1132.5 [767.5, 1400.0] | 1000.0 [712.5, 1625.0] | 0.97 |
| Chest drainage duration left,  median [IQR], d | 3.0 [2.0, 3.0] | 3.0 [2.0, 3.0] | 0.66 |
| Chest drainage duration right,  median [IQR], d | 3.0 [2.0, 3.0] | 3.0 [2.0, 4.0] | 0.31 |

Data are presented as median (with interquartile ranges) for continuous variables and n (%) for categorical variables. p values were obtained from Wilcoxon test for continuous variables, Fisher test for categorical variables, chi-square test for postoperative complications. Statistically significant p values (p < 0.05) are highlighted in bold.

An intraoperative video can be downloaded from: https://pan.baidu.com/s/1WDoD65BU72sJbaIen8u-eQ password: 60cj
